# Sex-specific effects of psychoactive pollution on behavioral individuality and plasticity in fish

**DOI:** 10.1093/beheco/arad065

**Published:** 2023-08-14

**Authors:** Giovanni Polverino, Upama Aich, Jack A Brand, Michael G Bertram, Jake M Martin, Hung Tan, Vrishin R Soman, Rachel T Mason, Bob B M Wong

**Affiliations:** School of Biological Sciences, Monash University, 25 Rainforest Walk, Clayton, 3800, Victoria, Australia; Department of Ecological and Biological Sciences, University of Tuscia, L.go dell'Università snc, Viterbo, 01100, Italy; School of Biological Sciences, Monash University, 25 Rainforest Walk, Clayton, 3800, Victoria, Australia; School of Biological Sciences, Monash University, 25 Rainforest Walk, Clayton, 3800, Victoria, Australia; Department of Wildlife, Fish, and Environmental Studies, Swedish University of Agricultural Sciences, SE-907 36, Umeå,Sweden; Department of Wildlife, Fish, and Environmental Studies, Swedish University of Agricultural Sciences, SE-907 36, Umeå,Sweden; Department of Zoology, Stockholm University, Svante Arrhenius väg 18b114 18, Stockholm, Sweden; School of Biological Sciences, Monash University, 25 Rainforest Walk, Clayton, 3800, Victoria, Australia; Department of Mechanical and Aerospace Engineering, New York University , 370 Jay Street, Brooklyn, 11201, NY, USA; School of Biological Sciences, Monash University, 25 Rainforest Walk, Clayton, 3800, Victoria, Australia; School of Life and Environmental Sciences, Deakin University, 221 Burwood Highway, Burwood, 3125, Victoria, Australia; School of Biological Sciences, Monash University, 25 Rainforest Walk, Clayton, 3800, Victoria, Australia

**Keywords:** animal personality, contamination, ecotoxicology, environmental change, fluoxetine, pharmaceutical pollution, sex differences, sexual dimorphism

## Abstract

The global rise of pharmaceutical contaminants in the aquatic environment poses a serious threat to ecological and evolutionary processes. Studies have traditionally focused on the collateral (average) effects of psychoactive pollutants on ecologically relevant behaviors of wildlife, often neglecting effects among and within individuals, and whether they differ between males and females. We tested whether psychoactive pollutants have sex-specific effects on behavioral individuality and plasticity in guppies (*Poecilia reticulata*), a freshwater species that inhabits contaminated waterways in the wild. Fish were exposed to fluoxetine (Prozac) for 2 years across multiple generations before their activity and stress-related behavior were repeatedly assayed. Using a Bayesian statistical approach that partitions the effects among and within individuals, we found that males—but not females—in fluoxetine-exposed populations differed less from each other in their behavior (lower behavioral individuality) than unexposed males. In sharp contrast, effects on behavioral plasticity were observed in females—but not in males—whereby exposure to even low levels of fluoxetine resulted in a substantial decrease (activity) and increase (freezing behavior) in the behavioral plasticity of females. Our evidence reveals that psychoactive pollution has sex-specific effects on the individual behavior of fish, suggesting that males and females might not be equally vulnerable to global pollutants.

## Teaser

Prozac is present in freshwater systems across the globe as the pharmaceutical contaminant fluoxetine. The effect of fluoxetine on aquatic species’ behavioral variability is not yet clear. We show that male guppies become more similar to each other after exposure to fluoxetine, and females become less flexible in their behavior. These sex-specific differences in response to fluoxetine can have a meaningful impact on their ability to survive in a changing world.

## INTRODUCTION

It is well established that animals differ from each other in their behavior. Individuals of the same population can vary in both their average behaviors (i.e., behavioral individuality; Sih et al. 2004; Réale et al. 2007) and in how they adjust their behavior over time and in response to environmental changes (i.e., behavioral plasticity; Dingemanse et al. 2010; Dingemanse and Wolf 2013). Such behavioral variation has significant implications for species ecology and evolution. In fact, behavioral differences among individuals are known to be heritable, consistent across ecological contexts, and can impact animal fitness (Dingemanse et al. 2002; Sih et al. 2004; Dochtermann et al. 2015; Araya-Ajoy and Dingemanse 2017). For instance, risk-averse individuals are more likely to survive longer than risk-prone ones, but at the cost of having lower access to food resources and mates (but see (Moiron et al. 2019). In general, animal populations with greater behavioral variability are more resilient, have higher population growth, and persist longer in the face of environmental changes, as seen in taxa as diverse as ants (*Temnothorax longispinosus*; Modlmeier et al. 2012) and Chinook salmon (*Oncorhynchus tshawytscha*; Carlson and Satterthwaite 2011). Indeed, phenotypic variability provides the adaptive potential for animal populations to survive, and even thrive, in a rapidly changing world (Wong and Candolin 2015). Conversely, a reduction in the magnitude of behavioral differences among individuals increases the risk of extinction for animal populations, as observed in wild sockeye salmon (*Oncorhynchus nerka*) populations monitored over five decades (Schindler et al. 2010). Therefore, behavioral individuality and plasticity can influence population persistence and stability in the long term (Réale et al. 2007; Dingemanse and Wolf 2013).

Sex is an important source of within-species variation in animal behavior and life history (Smith and Blumstein 2008; Schuett et al. 2010; Tarka et al. 2018). In many species, males and females allocate resources differently between reproduction and self-maintenance, leading to sexual dimorphism in behavioral and morphological traits (Parker 1979; Hedrick and Temeles 1989; Stearns 1992). Given the fundamental differences in their life-history strategies, males and females are also likely to exhibit different levels of behavioral (co)variation among and within individuals (Hämäläinen et al. 2018; Immonen et al. 2018). For instance, individuals that are more exploratory and risk prone should acquire more resources to grow faster and reach maturation earlier (Biro and Stamps 2008), and also have lower behavioral stress responses (Réale et al. 2010), especially in the sex with higher resource requirements. Drawing from sexual selection theory, recent work suggests a variety of mechanisms driving sex differences in behavioral individuality and plasticity. For instance, the greater male variability hypothesis suggests that trait variability among males should be higher than females, as sexual selection is generally stronger in males (Shields 1982) (but see (Harrison et al. 2022)). By contrast, the estrus-mediated variability hypothesis predicts higher plasticity in female traits arising from female hormonal changes during the reproductive cycle (see Beery and Zucker 2011 and references therein). As phenotypic variability is the raw material on which selection operates, understanding sex differences in trait variability is key to understanding their importance for the ecology and evolution of animal groups. Surprisingly, however, little is known about sex differences in trait variability and their ecological importance (Zajitschek et al. 2020), especially in the face of rapid environmental change.

Pharmaceutical pollution is a global challenge that impacts ecologically relevant behaviors in wildlife (Montiglio and Royauté 2014; Bertram et al. 2022). Among pharmaceuticals, psychoactive drugs are particularly concerning as they act on evolutionarily conserved receptors that regulate various physiological and behavioral systems shared by both target and non-target species alike (Fent et al. 2006), and persist in the aquatic ecosystems over long time periods (Kümmerer et al. 2018). Therefore, it should come as no surprise that psychoactive contaminants can significantly alter fundamental behaviors of animals, including activity and stress-related behaviors (Brodin et al. 2017; Martin et al. 2017; Sehonova et al. 2018). In health research, the effects of psychoactive drugs on men are far better understood than on women, since the latter are typically underrepresented in experimental trials (Nowogrodzki 2017). As a result, there are reported cases of women being inadvertently overmedicated because receiving the same dosage of medications prescribed to men (Zucker and Prendergast 2020). Likewise, psychoactive pollutants can have sex-specific collateral (average) effects on animal behavior (Lisboa et al. 2007; Gobinath et al. 2016; Saaristo et al. 2017; Thoré et al. 2021). Surprisingly, to date, only a very limited number of studies have investigated the long-term impacts of psychoactive pollutants on behavioral variability in animal populations (Tan et al. 2020; Polverino et al. 2021; Henry et al. 2022), and none have specifically examined sex-specific effects. Thus, it remains unclear whether and to what extent sexes differ in their response to chronic psychoactive pollution, and whether such differences might impact the phenotypic variability of animal populations as a whole.

Here, we propose that selective pressures originating from life-history and reproductive investments can result in sex-specific vulnerabilities to a pervasive psychoactive pollutant of global concern. To do so, we exposed fish to fluoxetine (Prozac), a widely prescribed selective serotonin reuptake inhibitor and pharmaceutical pollutant detected in aquatic environments worldwide, with concentrations in surface waters ranging from < 1 to 350 ng L^−1^ (Schultz et al. 2010; Weinberger and Klaper 2014; Tan et al. 2020). We extend prior analyses on the effects of long-term exposure to fluoxetine (Polverino et al. 2021)﻿ to test whether effects among and within individual guppies (*Poecilia reticulata*) are sex-specific. It has previously been established that exposure to antidepressants can reduce among-individual variation in the behavior of these fish (Polverino et al. 2021), potentially impairing the adaptive potential of entire populations. However, whether males and females are equally vulnerable to these pollutants remains unknown. In guppies, males exhibit alternative mating strategies, and females can mate with multiple males within a reproductive cycle. As a result, lifetime investments differ between males and females, potentially contributing to the evolution and maintenance of variation in their behavioral traits (Schuett et al. 2010). Sexes can also differ in their sensitivities to drugs, often due to sex-specific differences in the metabolic and absorptive processes (Soldin and Mattison 2009). Therefore, we predict that exposure to environmentally realistic levels of a psychoactive pollutant, such as fluoxetine, would have sex-specific effects on the behavioral variation of fish. By exposing wild-caught fish to the pollutant for 2 years (six generations), we could then examine the potential effects of long-term exposure to fluoxetine on sex-specific behaviors at the individual level.

## METHODS

### Multigenerational exposure

We used the guppy as our model species, since they are known to inhabit freshwater systems impacted by pharmaceutical contamination (Widianarko et al. 2000; Araújo et al. 2009). Fish were sourced from a long-term mesocosm system founded by wild-caught individuals. Briefly, 3600 sexually mature wild guppies were collected from Alligator Creek (19°23ʹ50.3ʹʹ S, 146°56ʹ56.5ʹʹ E), which is free from fluoxetine contamination (Tan et al. 2020). Adult fish (*n* = 300; 50:50 sex ratio) were randomly assigned to one of 12 independent mesocosms (180 × 60 × 60 cm; water depth: 30 cm; 648 L) simulating shallow, vegetated aquatic habitats. Mesocosms were maintained in a temperature-controlled facility (23.4 ± 1.0 °C) under a 12:12 h light:dark cycle and filled with aerated, carbon-filtered freshwater. Mesocosms were also supplied with a gravel substrate and natural vegetation (Java moss, *Taxiphyllum barbieri*) to simulate the guppies’ natural habitat. Fish were fed until satiation with commercial fish food every second day (Aquasonic Nutra Xtreme C1), while 20% water changes were performed weekly.

**Figure 1:**
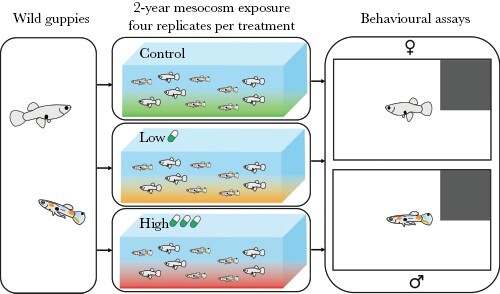
Schematic of the experimental design. Adult guppies with an equal sex ratio were collected from the wild and exposed to control, low, and high fluoxetine concentrations across 12 replicated mesocosms (180 × 60 × 60 cm). After 2 years of exposure, males and females from each exposure treatment (four independent mesocosms per treatment) were behaviorally phenotyped in open-field arenas (25 × 15 × 15 cm).

After 5 months of acclimation in the mesocosms, fish were exposed to fluoxetine. The full details of mesocosm establishment, fluoxetine exposure, and experimental protocols have been reported elsewhere (Tan et al. 2020; Polverino et al. 2021). Briefly, fish were randomly assigned to one of three exposure treatments (*n* = 4 independent mesocosm populations per treatment): control (mean ± SE = 0 ± 0 ng L^−1^), low fluoxetine (40 ± 3 ng L^−1^), and high fluoxetine (366 ± 28 ng L^−1^) for two consecutive years (i.e., 24 months; Figure 1). The low- and high-fluoxetine treatments reflect concentrations repeatedly detected in freshwater habitats, with the former representing common surface water concentrations in fluoxetine-contaminated systems and the latter representing levels typically found in heavily effluent-dominated waterbodies (Mole and Brooks 2019). The semi-natural mesocosms set-up with three environmentally realistic levels of fluoxetine exposure allowed us to study the long-term, sex-specific effects of this global pollutant on the behavioral phenotypes of guppies. The details of dosing and analytical verification (using HPLC-MS-MS) of fluoxetine treatment levels are summarized in the Supplementary Material.

### Experimental fish

We provide here a synopsis of the experimental protocol detailed in (Polverino et al. 2021). We assayed the behavior of 120 sexually mature fish (five females and five males per mesocosm). Fish were randomly captured from their population mesocosms and individually transferred into glass holding tanks (12 × 23 cm, diameter × height) filled with 2 L of treatment water from their native mesocosm (i.e., pollutant exposure was maintained throughout testing). Each holding tank was aerated, contained a gravel substrate (2 cm layer) and live vegetation (Java moss), and was covered on all sides to reduce external disturbance. Water temperature (24 ± 1.0 °C) was monitored daily, and fish were kept on a 12:12 h light:dark cycle. Fish were fed until satiation as in their native mesocosm populations, and were acclimated to the individual holding tanks for 48 h before behavioral assays commenced.

### Behavioral assays

We ran an open-field assay validated for studying individual-level variation in the behavior of guppies (Burns 2008). Before the behavioral trial started, each fish was placed into an opaque plastic cylinder and allowed to acclimate for 2 min. Individual fish were then carefully placed into an open-field arena (25 × 15 × 15 cm). Each arena contained a white background with a dark, square region (7.5 × 7.5 cm) in one corner that served as a refuge. The presence of the refuge ensured that fish had a safe area accessible when they chose to explore the novel and potentially dangerous open space (see Figure 1 and Polverino et al. 2021 for details). The arenas were filled with treatment water from the respective mesocosms, to ensure that fluoxetine exposure was maintained for each fish throughout the experiment. Water was replaced in each arena between consecutive trials to eliminate the potential build-up of conspecific cues.

Upon entering the arena, each fish was allowed to explore freely for 20 min, and its behavior was filmed from above with a high-resolution camera (Panasonic HC-V180). Following the completion of the trial, the fish was returned to its individual holding tank. This process was repeated four times for each fish (i.e., four repeated measures), with 3 days between consecutive trials. Experimental videos were automatically tracked using EthoVision XT v. 14.0.1326 (Noldus Information Technologies), blind to the treatment.

We chose mean velocity as a measure of fish activity (the total distance moved by an individual divided by the time spent moving, in seconds), since it captures two main and often correlated activity-related traits: distance moved and time spent moving. Freezing behavior (immobility, in seconds) was adopted as a standard proxy for fish stress response (Cachat et al. 2010; Polverino et al. 2022). Individual variation in these traits is a key target of selection in all non-sessile animals (Réale et al. 2007), and is known to have ecological and evolutionary implications (Wolf and Weissing 2012). We followed standard experimental protocols (Polverino et al. 2021) that have been successfully used to investigate these behavioral traits (Polverino et al. 2016, 2018).

After completing all behavioral trials, we measured the standard body length of each fish (i.e., from the tip of the snout to the caudal peduncle;  ± 0.01 mm) to statistically account for any behavioral variation explained by size.

### Statistical analyses

Of the 120 fish used, 116 individuals completed all four behavioral trials (control: 20 females and 20 males; low fluoxetine: 18 females and 19 males; high fluoxetine: 19 females and 20 males), resulting in a total of 469 observations included in the analysis (i.e., over 156 h of video recordings).

We analyzed data using Bayesian mixed-effects models fitted with the *brms* package (Bürkner 2017) in *R* v. 4.2.1 (R Core Team 2022). We ran generalized linear mixed-effects models to test for possible sex-specific effects of fluoxetine exposure on fish behavior. Models were run for 6000 iterations (1000 warmups) on four chains, using a thinning interval of 2 (total post-warmup samples = 10,000). We used weakly informative normal priors (*N* (0,10)) for fixed effects and positively bound exponential (1) priors for random effects. However, models were also run using default priors to confirm that results were robust to prior specifications. We performed posterior predictive checks to ensure adequate model fits, while trace plots confirmed that models converged with low among-chain variability (Rhat = 1.00). We report posterior means with 95% credible intervals (CIs) for all parameter estimates, where inference was based on CIs that did not include zero.

To test whether fluoxetine exposure had sex-specific effects on fish behavior, at both the average and individual levels, we fitted separate univariate models with mean velocity and freezing behavior as the response variables. Mean velocity and freezing behavior were scaled (mean = 0, SD = 1) to aid in model fitting and interpretation. We assumed a Gaussian error distribution, which was confirmed after inspection of the model residuals. Each model included the interaction between sex and treatment as fixed factors, while trial (four repeated measures per individual), standard length, and time spent outside the refuge were added as covariates. Both standard length and time spent outside the refuge actively exploring the open field were mean-centered (mean = 0, SD = 1), and trial was left-centered (i.e., trial 1 = 0, to set the model intercept at the first trial) to aid in model fitting. We added mesocosm (*n* = 12; four per treatment) as a random intercept in our model. Individual ID was also included as a random intercept in the models, separately for each sex and treatment combination. We also included a sex-by-treatment interaction in the residual portion of the model to estimate the within-individual (residual) variance for each treatment-sex combination. This model structure allowed us to test for the effects of fluoxetine exposure on average behaviors, as well as among-individual (*V*_A_) and residual within-individual behavioral variance (*V*_W_) in males and females separately (Chapple et al. 2022) (see model structure in Supplementary Table S1). Further, we calculated the magnitude difference (Δ*V*) in both the among- (Δ*V*_A_) and within-individual (Δ*V*_W_) variance to test whether these behavioral components varied between males and females from each exposure treatment (Royauté and Dochtermann 2021). The Bayesian framework allowed us to directly estimate the distribution of Δ*V*s by estimating the difference in the posterior distributions of two separate variance components. The posterior mean of Δ*V* can, therefore, be interpreted as the estimated strength of Δ*V*, with 95% CIs representing the precision around this estimate (Royauté and Dochtermann 2021).

To examine whether among- and within-individual correlations between mean velocity and freezing behavior differed across treatments, we ran separate bivariate linear mixed-effects models for each treatment and sex, in which both mean velocity and freezing behavior were included as response variables. We included individual ID as a random intercept to calculate treatment-specific correlations between activity and freezing behavior. Similar to the univariate models above, we ran the bivariate models on four chains for 6000 iterations (1000 warmup), using weakly-informative normal priors (*N* (0,10)) for fixed effects, and positively-bound exponential (1) priors for random effects.

All results of the models run with default priors were qualitatively similar to those reported in the main text (see Supplementary Tables S4 and S5 for model output).

## RESULTS

### Effects among and within individuals

#### Among-individual variance (behavioral individuality)

**Figure 2:**
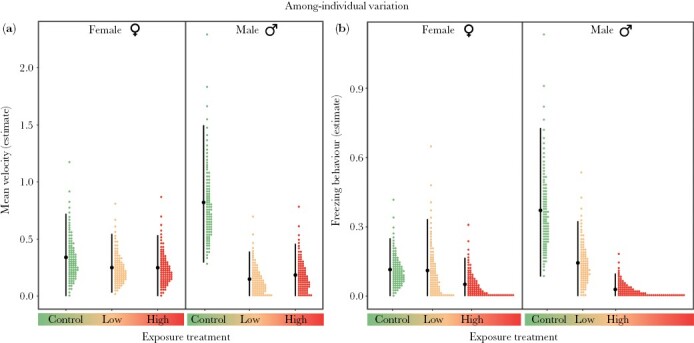
Among-individual variance in (a) activity (mean velocity, cm per second) and (b) stress response (freezing behavior, in seconds) of female and male fish from the exposure treatments (control, low fluoxetine, and high fluoxetine). In each plot, filled-black circles represent the mean variance estimates, vertical error bars denote 95% credible intervals, and colored-dotted values represent probability density.

Fluoxetine exposure had sex-specific effects on activity and stress response, resulting in lower among-individual variation in males (i.e., lower behavioral individuality) but not in females (Table 1). More specifically, males from the control treatment differed more from each other in their activity compared to those from the low- and high-fluoxetine treatments (Table 1a; Figure 2a). However, there was some uncertainty around the difference between controls and high-fluoxetine fish, with CIs slightly overlapping with zero (Δ*V*_A_ [95% CI] = 0.635 [−0.057, 1.384]; Table 1a; Figure 2a). Among-individual variation in activity did not differ between males from the low- and high-fluoxetine treatments, revealing a non-monotonic effect of fluoxetine on males (Table 1a; Figure 2a). We also found comparable effects of fluoxetine on the stress response of guppies, with males from the control treatment differing more among each other than males exposed to high fluoxetine levels (Table 1a; Figure 2b). However, no differences in among-individual variance in stress response were observed between males from the control and low-fluoxetine treatments, or those from the low- and high-fluoxetine treatments (Table 1a; Figure 2b).

**Table 1:** The effect size (±95% CI) of the magnitude difference in (a) among-individual variation (behavioral individuality; Δ*V*_A_) and (b) within-individual variation (behavioral plasticity; Δ*V*_W_) of activity (mean velocity, in cm per second) and stress response (freezing behavior, in seconds) in male and female fish from each exposure treatment (control, low, and high fluoxetine)

Among-individual variation (Δ*V*_A_)			
Behavior	Treatment contrast	Female	Male
*Activity*	Control-low	0.090 (−0.404, 0.607)	**0.672 (0.026, 1.430)**
	Control-high	0.091 (−0.410, 0.612)	0.635 (−.057, 1.384)
	High-low	−0.001 (−0.437, 0.416)	0.036 (−0.354, 0.430)
*Stress response*	Control-low	0.004 (−0.300, 0.256)	0.227 (−0.141, 0.664)
	Control-high	0.064 (−0.117, 0.258)	**0.342 (0.045, 0.719)**
	High-low	−0.060 (−0.341, 0.178)	−0.115 (−0.321, 0.068)
Within-individual variation (Δ*V*_W_)			
Behavior	Treatment contrast	Female	Male
*Activity*	control-low	**0.402 (0.099, 0.728)**	−0.065 (−0.354, 0.205)
	Control-high	0.157 (−0.210, 0.530)	−0.309 (−0.651, 0.043)
	High-low	0.245 (−0.005, 0.520)	0.243 (−0.102, 0.626)
*Stress response*	Control-low	−**0.241 (**−**0.432,** −**0.058)**	0.033 (−0.198, 0.258)
	Control-high	−**0.246 (**−**0.434,** −**0.064)**	0.145 (−0.043, 0.338)
	High-low	0.005 (−0.235, 0.252)	−0.112 (−0.293, 0.084)

Contrasts in bold with 95% CIs are those that did not overlap with zero.

In sharp contrast, fluoxetine exposure did not affect the among-individual variance in activity or stress response of females (Table 1; Figure 2).

#### Within-individual variance (behavioral plasticity)

**Figure 3:**
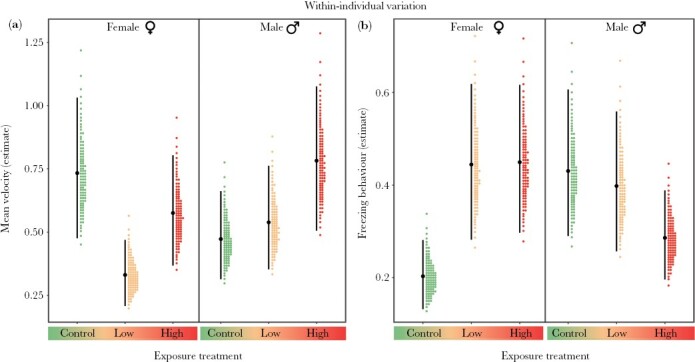
Within-individual variance in (a) activity (mean velocity, cm per second) and (b) stress response (freezing behavior, in seconds) of female and male fish from the exposure treatments (control, low fluoxetine, and high fluoxetine). In each plot, filled-black circles represent the mean variance estimates, vertical error bars denote 95% credible intervals, and colored-dotted values represent probability density.

Fluoxetine exposure also had sex-specific effects on within-individual behavioral variance (i.e., behavioral plasticity) but in the opposite direction to what was observed among individuals: effects were strong in females, but negligible in males (Table 1b). Compared to control females, exposure to fluoxetine resulted in lower within-individual variance in the activity of low-treatment females but not females exposed to high levels of the drug (Table 1b, Figure 3a). The within-individual variance in female activity tended to decline from the high to the low fluoxetine treatment, although CIs marginally overlapping with zero indicated that there was some uncertainty around this evidence (Δ*V*_W_ [95% CI] = 0.245 [−0.005, 0.520]; Table 1b). With respect to stress response behavior, control females had lower within-individual variance than females exposed to either the low or high fluoxetine levels, while low- and high-fluoxetine females did not differ from each other, revealing a non-monotonic effect of fluoxetine on females (Table 1b; Figure 3b).

On the contrary, within-individual variation in male activity levels and stress response did not differ across treatments (Table 1b, Figure 3a), except for a marginal, weak difference in the stress response with higher values observed in control than high-fluoxetine males (Δ*V*_W_ [95% CI] = 0.145 [−0.043, 0.338], also see Table 1b; Figure 3b).

#### Behavioral correlations

We found evidence of treatment- and sex-specific correlations between activity and stress response, both at the among- and within-individual levels (Table 2). In the control treatment, activity and stress response were not correlated either among or within individuals, except for a moderate, positive within-individual correlation observed in females (Table 2). However, in the low-fluoxetine treatment, we found evidence of strong among-individual correlations between activity and stress response in both sexes, and a moderate within-individual correlation among males, but not females. Activity and stress response were not correlated either among or within individuals in the high-fluoxetine treatment (Table 2).

**Table 2:** Correlation estimates (among and within individuals) for activity (mean velocity, in cm per second) and stress response (freezing behavior, in seconds) across the exposure treatments (control, low, and high fluoxetine)

Correlations	Treatment	Female	Male
*Among-individual (V*_*A*_)	Control	0.12 (−0.57, 0.69)	0.03 (−0.47, 0.54)
	Low fluoxetine	**0.60 (0.13, 0.91)**	**0.58 (0.02, 0.92)**
	High fluoxetine	0.21 (−0.61, 0.85)	0.14 (−0.70, 0.84)
*Within-individual (V*_*W*_)	Control	**0.44 (0.21, 0.63)**	0.07 (−0.18, 0.32)
	Low fluoxetine	0.07 (−0.19, 0.33)	**0.33 (0.09, 0.55)**
	High fluoxetine	−0.03 (−0.27, 0.22)	0.02 (−0.22, 0.26)

Estimates of correlation coefficients with 95% credible intervals are represented for each treatment. Bold values correspond to correlation coefficients whose confidence intervals do not overlap with zero.

### Mean-level effects

Our findings do not support either an interactive or independent effect of sex and fluoxetine exposure on the average activity and stress response of the fish (Supplementary Table S2). Likewise, standard length did not have a detectable effect on activity or stress response behavior. However, trial number had a negative effect on activity levels, with fish decreasing their average activity as trials progressed (Supplementary Table S2). In contrast, trial number had a weak, positive effect on stress response, with fish freezing more over successive trials (estimate ± 95%CI: 0.05 [0.00, 0.09]). As expected, when fish spent more time outside of the refuge, their average activity increased, and they had more opportunities to display stress responses (Supplementary Table S2).

## DISCUSSION

The collateral effects of pharmaceutical pollution on wildlife are of growing concern, having the potential to disrupt population dynamics and ecosystem functioning (Arnold et al. 2014; Saaristo et al. 2018). Here, we provide empirical evidence that chronic exposure to a globally pervasive psychoactive pollutant has a sex-specific effect on individual variation in the behavior of fish. Specifically, we found that exposure to fluoxetine reduced variation among males (lower behavioral individuality) in both activity and stress response, while no impact was observed on the behavioral individuality of females. In sharp contrast, exposure to the drug had opposite sex-specific effects at the within-individual level, altering the behavioral plasticity of females but not males. Our results reveal novel pathways through which psychoactive pollutants can impact the phenotypic variability of animal populations, highlighting that males and females are not equally vulnerable to this widespread pollutant.

Our key finding is that long-term exposure to fluoxetine affects the behavioral individuality of males, but not females, by reducing among-individual variation in their activity and stress response behaviors—two independent behavioral axes. This evidence expands the knowledge that fluoxetine exposure reduces behavioral differences among fish in general (Tan et al. 2020; Polverino et al. 2021), revealing effects that manifest primarily in males. This sex-specific loss in behavioral variability, therefore, suggests that variation among male guppies is more vulnerable to psychoactive pollutants than variation among females. Greater variance in reproductive success (Janicke et al. 2016) and phenotypic traits such as morphology and behavior (Wyman and Rowe 2014) is, in fact, especially crucial for males throughout the animal kingdom since sexual selection on males is typically more intense, according to the “greater male variability hypothesis” (Zajitschek et al. 2020), but also see (Harrison et al. 2022). For instance, less competitive males can adopt alternative mating strategies and take more risks to secure mating compared to those that are more competitive and preferred by females (Gross 1996). A reduction in diverse behavioral strategies among males due to fluoxetine exposure is likely to decrease the fitness advantages of such strategies and gradually collapse such variability among individuals, as reported in fish exposed to various selective pressures from their environment (Bell and Sih 2007; Dingemanse et al. 2007) and anticipated for wildlife under anthropogenic disturbance (Geffroy et al. 2020). This is especially important because fluoxetine, like numerous other pharmaceutical pollutants, is continually discharged in effluent from wastewater treatment plants (Mole and Brooks 2019) and is relatively stable (Kwon and Armbrust 2006), causing long-term contamination of aquatic ecosystems. Our result highlights the significant threat posed by fluoxetine to key evolutionary processes, including sexual selection, and ultimately to the persistence of wild populations in polluted habitats (Arnold et al. 2014; Saaristo et al. 2018).

Our evidence also reveals that chronic exposure to this widespread pollutant altered the behavioral plasticity of females but not males, shrinking their within-individual variation in activity while increasing such variation in their stress behavior. Similar results have also been found in aquatic snails (*Physa acuta*, Henry et al. 2022) and hermit crabs (*Pagurus bernhardus*, Nanninga et al. 2020), in which exposure to environmental pollutants—including fluoxetine—reduced plasticity in the activity levels of the individuals. Since maintaining the sensory and regulatory machinery necessary for high responsiveness is costly for an organism (reviewed in DeWitt et al. 1998), reducing plasticity may be interpreted as an adaptive strategy to budget energy across competing functions (Westneat et al. 2015). This becomes especially critical for female guppies living in contaminated habitats, given their higher resource requirements relative to males.

Yet female guppies increased their plasticity in stress–response behavior under fluoxetine exposure. Although counterintuitive, this result may not be surprising given the role of fluoxetine (Prozac) as a potent anxiolytic (Rossi et al. 2004). It is possible that exposure to fluoxetine reduced the consistency of stress responses, which is typically observed in some individuals of a population (i.e., on one side of the continuum), resulting in less predictable behaviors in those individuals. This effect should be especially noticeable in females, in which phenotypic plasticity *per se* is more pronounced than in their male counterparts according to the “estrus-mediated variability hypothesis” (Zajitschek et al. 2020). An alternative explanation is that for females it may be beneficial, rather than costly, to be unpredictable in their risk avoidance under disturbed environments (Westneat et al. 2015). In fact, classic work predicts that behavioral plasticity increases in prey animals under potentially dangerous conditions (Hugie 2003; Briffa 2013), since unpredictable behaviors reduce vulnerability to predation (Stamps et al. 2012). This should be particularly relevant for female guppies, given their life history and size which make them a greater target for predation than males. Under this perspective, females from disturbed environments should invest more in reducing their vulnerability by adopting less-predictable behavioral strategies when tested in novel and potentially dangerous open spaces. Overall, we report that a global pharmaceutical pollutant triggers substantial changes in the plasticity of ecologically relevant behaviors in female fish, which are likely to impact their adaptive strategies to survive in a rapidly changing world.

From a mechanistic perspective, sex differences in the behavior of guppies observed in our study could be explained by potential differences between males and females in their neurophysiological responses to the psychoactive drug. As a selective serotonin reuptake inhibitor, fluoxetine directly modulates the functions of the serotonergic system, which is known to play a crucial role in regulating the behavior of animals, including fish (Ferreira et al. 2023). The serotonergic system often differs between the sexes (i.e., in receptor distribution, neurotransmitter synthesis, and serotonin transporter expression [Zucker and Prendergast 2020; Ferreira et al. 2023]), and this, in turn, could underpin the sex differences in behavior observed. While identifying the exact mechanism was beyond the scope of the current study, further investigation into the neurobiological pathways and molecular mechanisms will enhance our understanding of the sex-specific effects of fluoxetine and their implications for wildlife ecology and evolution.

We also found some non-monotonic responses to fluoxetine in both male and female fish, meaning that the relationships between fluoxetine dosage and fish response were not necessarily linear (Vandenberg et al. 2012). This pattern has been previously reported as a characteristic of fluoxetine’s mode of action on the behavioral traits of a large number of animal species (Barry 2013; Martin et al. 2017; Saaristo et al. 2017; Tan et al. 2020). Broadly, such non-monotonic dose–response relationships could result from several different mechanisms, such as receptor desensitization, negative feedback with increasing dose, dose-dependent metabolism modulation, and/or opposing effects induced by an analyte binding to multiple receptors that differ in their affinity (Vandenberg et al. 2012). The exact mechanism(s) behind fish non-monotonic responses to fluoxetine is/are beyond the scope of this work. Nevertheless, this evidence has important ecological implications, as it suggests that exposures to even very low concentrations can alarmingly have large effects on the phenotypic variability of wildlife (Polverino et al. 2021).

In our study, the two measured behavioral traits—mean velocity and freezing behavior—were generally uncorrelated at the individual level, confirming that they represented two separate behavioral axes. However, a behavioral syndrome emerged in individuals exposed to low fluoxetine concentrations (Sih et al. 2004), suggesting that under fluoxetine exposure, the variation among individuals in one trait predicted their variation in the other. It remains unknown whether these effects are permanent or can be reversed after the withdrawal of the contaminant. A large body of literature indicates that chronic exposure to fluoxetine leads to changes in neuronal morphology and activity that impair behavior (Galea et al. 2008) and that such changes could transcend generations (Gobinath et al. 2016). This suggests that even temporary contamination of habitats may have long-lasting effects on fish populations, including guppies (Tan et al. 2020).

The average effects of psychoactive drugs on animal behavior can differ between males and females (Lisboa et al. 2007; Gobinath et al. 2016; Saaristo et al. 2017; Thoré et al. 2021). Yet we found no significant effects of fluoxetine on the mean-level behaviors of either male or female fish. This aligns with previous research on multigenerational exposure in a range of animal taxa, which found no clear effects of fluoxetine on mean-level animal behaviors (e.g., guppies (Tan et al. 2020), snails (Henry et al. 2022), and water fleas (Heyland et al. 2020)). However, it is important to note that the average effects of fluoxetine are not always consistent across species and even behavioral traits (Martin et al. 2017; Saaristo et al. 2017), and can vary depending on the exposure period and dosage (De Castro-Català et al. 2017). Our evidence highlights the importance of considering individual-level and sex-specific effects when studying the impact of global contaminants on aquatic organisms, since consequences can extend beyond the better-known effects at the mean level.

In conclusion, we offer empirical evidence that the effect of a global pharmaceutical pollutant on the behavioral individuality and plasticity of wildlife can differ between the sexes. Specifically, our work reveals that exposure to fluoxetine decreases variation in the behavioral individuality of males, but not females. On the contrary, fluoxetine exposure affects behavioral plasticity in females, but not males. The substantial loss of phenotypic diversity in males and changes in the plasticity levels of females indicate that even low doses of this widespread pollutant can have large repercussions on the ecology and evolution of wildlife, ultimately threatening the resilience of animal populations and their adaptive capacity to survive in an increasingly contaminated world. Developing a better understanding of the sex-specific effects of global pollutants on the phenotypic variability and plasticity of animal populations is an essential goal for future research.

## Supplementary Material

arad065_suppl_Supplementary_Material

## Data Availability

Analyses reported in this article can be reproduced using the data provided by Polverino et al. (2023).

## Notes

We thank David Williams and Envirolab Services for analytical testing of water samples.
