# Supplementary material for: Sex-specific effects of psychoactive pollution on behavioral individuality and plasticity in fish
Source: Behav Ecol. 2023 Aug 14;34(6):969–78. doi: 10.1093/beheco/arad065 (PMC10636733; doi:10.1093/beheco/arad065)
Supplement: arad065_suppl_Supplementary_Material [file arad065_suppl_supplementary_material.docx]

**SUPPLEMENTARY MATERIAL**

Sex-specific effects of psychoactive pollution on behavioural individuality and plasticity in fish

Giovanni Polverino†, Upama Aich†, Jack A. Brand, Michael G. Bertram, Jake M. Martin, Hung Tan, Vrishin R. Soman, Rachel T. Mason, Bob B. M. Wong

† Joint first authors

**Methods**

**Fluoxetine dosing and analytical verification of treatment levels**

Nominal fluoxetine concentrations in the mesocosm tanks were maintained via static renewal. For both fluoxetine treatments (low and high), mesocosm tanks were dosed twice weekly (as described in detail in [1,2]). Briefly, this process involved preparing two separate stock solutions with either 2 mg or 20 mg of fluoxetine hydrochloride (Sigma Aldrich; product number: F132, CAS: 56296-78-7) dissolved in 100 mL of methanol, for the low and high treatments, respectively. Stock solutions were diluted to produce dosing solutions: 1 mL aliquot of stock solution (low or high) was diluted with 1,000 mL of reverse osmosis water. Further, to control for any potential solvent effects and maintain consistent levels of handling across all mesocosm tanks, a solvent solution (1 mL of methanol in 1000 mL of reverse osmosis water) was added to all unexposed tanks twice per week. Therefore, all 12 mesocosm tanks received 2 mL of methanol weekly (0.0006% per mesocosm water volume).

To verify concentrations of fluoxetine over the two-year exposure period, water samples (40 mL) were drawn from all mesocosm tanks in the low and high treatments once a month. To ensure the absence of fluoxetine contamination in control tanks (unexposed treatment), water samples were also collected from these mesocosms every two months throughout the study. Water samples were stored at 4 °C in the dark and analysed with gas chromatography–tandem mass spectrometry (7000C Triple Quadrupole GC-MS/MS, Agilent Technologies, Delaware, USA; limit of quantification: 2 ng L^−1^). Water analysis was performed by Envirolab Services (MPL Laboratories; NATA accreditation: 2901; accredited for compliance with ISO/IEC: 17025). A detailed description of the water analysis protocol is in [3].

**Statistical analyses**

**Table S1.** **Examples of linear mixed-effect model syntax used for the statistical analysis.** We used univariate models to measure sex-specific, treatment-wise individual behavioural variation and mean effects. Bivariate models were used to examine among- and within-individual correlations between activity and stress response for each sex per treatment. Response variables and covariates were scaled to aid in model fitting and interpretation. Models are written following *R* language: response variable ~ fixed factors + covariates + (random slopes | |random intercept) using *brms* package.

| **Aim** | **Analysis** | **Model structure** |
| --- | --- | --- |
| To test for effects of long-term fluoxetine exposure on sex-specific behaviours at both average and individual levels | Univariate model | Example for activity measure:  activity <- bf (mean velocity~ sex*treatment + trial +refuge use+ body size +(0+sex\| \|gr (ID, by = treatment)) + (1\|mesocosm), sigma ~ 0+sex: treat, family = gaussian)  This model structure was then repeated for measuring stress response. |
| To test among- and within-individual correlations between activity and stress response | Bivariate model | Example of behavioural correlation in control females:  female activity <- bf (mean velocity ~ (1\|a\|gr(ID, by = control_treatment)), family = gaussian) +  female stress response <- bf (scale (freezing behaviour) ~ (1\|a\|gr (ID, by = control_treatment)), family = gaussian)  This model structure was then repeated for measuring each sex by treatment combination. |

**Results**

**Table S2**. **Output from Bayesian linear mixed-effects models investigating the effects of different fluoxetine treatments in females and males for (a) activity (mean velocity, in cm per second) and (b) stress response (freezing, in seconds).** Bold estimates with 95% credible intervals indicate fixed effects and intercept values that differ from zero. Sex (female) and treatment (control) are the reference categories for fixed effects. Random effects are presented in standard deviation (SD) units and random variances in log SD units.

| Behaviour | Predictors | Estimate (95% CI) |
| --- | --- | --- |
| *Activity* |  |  |
| Fixed effects |  |  |
|  | Intercept | 0.41 (-0.04, 0.85) |
|  | sex (male): treatment (low fluoxetine) | 0.39 (–0.28, 1.04) |
|  | sex (male): treatment (high fluoxetine) | 0.37 (–0.31, 1.05) |
|  | sex (male) | –0.23 (–0.82, 0.36) |
|  | treatment (low fluoxetine) | –0.39 (–0.97, 0.19) |
|  | treatment (high fluoxetine) | –0.27 (–0.85, 0.32) |
|  | trial | –**0.14 (**–**0.20,** –**0.08)** |
|  | refuge use | **0.23 (0.15, 0.32)** |
|  | body size | –0.20 (–0.34, –0.06) |
| Random intercepts |  |  |
|  | mesocosm | **0.26 (0.04, 0.55)** |
| *sex: treatment* | |  |
|  | female: control | **0.56 (0.23, 0.91)** |
|  | female: low fluoxetine | **0.48 (0.23, 0.79)** |
|  | female: high fluoxetine | **0.48 (0.18, 0.79)** |
|  | male: control | **0.89 (0.60, 1.29)** |
|  | male: low fluoxetine | **0.35 (0.03, 0.69)** |
|  | male: high fluoxetine | **0.39 (0.05, 0.74)** |
| Residual variances |  |  |
| *sex: treatment* | |  |
|  | female: control | –0.17 (–0.34, 0.03) |
|  | female: low fluoxetine | –**0.56 (**–**0.75,** –**0.35)** |
|  | female: high fluoxetine | –**0.29 (**–**0.47,** –**0.09)** |
|  | male: control | –**0.38 (**–**0.56,** –**0.19)** |
|  | male: low fluoxetine | –**0.32 (**–**0.50,** –**0.12)** |
|  | male: high fluoxetine | –0.13 (–0.31, 0.06) |
|  |  |  |
| *Stress response* | | |
| Fixed effects |  |  |
|  | Intercept | -0.00 (-0.66, 0.66) |
|  | sex (male): treatment (low fluoxetine) | 0.19 (–0.31, 0.70) |
|  | sex (male): treatment (high fluoxetine) | 0.33 (–0.13, 0.78) |
|  | sex (male) | –0.14 (–0.56, 0.27) |
|  | treatment (low fluoxetine) | 0.03 (–0.94, 0.88) |
|  | treatment (high fluoxetine) | –0.26 (–1.19, 0.65) |
|  | trial | **0.05 (0.00, 0.09)** |
|  | refuge use | **0.69 (0.62, 0.75)** |
|  | body size | –0.09 (–0.21, 0.02) |
| Random intercepts |  |  |
|  | mesocosm | **0.59 (0.36, 1.00)** |
| *sex: treatment* | |  |
|  | female: control | **0.32 (0.13, 0.55)** |
|  | female: low fluoxetine | **0.29 (0.02, 0.64)** |
|  | female: high fluoxetine | **0.19 (0.01, 0.45)** |
|  | male: control | **0.59 (0.35, 0.91)** |
|  | male: low fluoxetine | **0.36 (0.09, 0.62)** |
|  | male: high fluoxetine | **0.14 (0.01, 0.35)** |
| Residual variances |  |  |
| *sex: treatment* | |  |
|  | female: control | –**0.81 (**–**0.98,** –**0.61)** |
|  | female: low fluoxetine | –**0.42 (**–**0.61,** –**0.22)** |
|  | female: high fluoxetine | –**0.41 (**–**0.59,** –**0.23)** |
|  | male: control | –**0.43 (**–**0.61,** –**0.24)** |
|  | male: low fluoxetine | –**0.47 (**–**0.66,** –**0.27)** |
|  | male: high fluoxetine | –**0.63 (**–**0.80,** –**0.46)** |

**Table S3.** **Among- and within-individual variance estimates with 95% credible intervals in activity (mean velocity, in cm per second) and stress response (freezing behaviour, in seconds) in males and females exposed to different fluoxetine treatments.**

| *Among-individual variance* | | | |
| --- | --- | --- | --- |
| Behaviour | **Fluoxetine Treatment** | **Female** | **Male** |
| *Activity* | control | 0.340 (0.000, 0.721) | 0.820 (0.295, 1.498) |
|  | low fluoxetine | 0.250 (0.031, 0.546) | 0.149 (0.000, 0.391) |
|  | high fluoxetine | 0.249 (0.000, 0.535) | 0.185 (0.000, 0.459) |
|  | | | |
| *Stress response* | control | 0.115 (0.000, 0.251) | 0.371 (0.085, 0.728) |
|  | low fluoxetine | 0.111 (0.000, 0.333) | 0.144 (0.000, 0.324) |
|  | high fluoxetine | 0.051 (0.000, 0.166) | 0.029 (0.000, 0.098) |
|  |  |  |  |
| *Within-individual variance* | | | |
| Behaviour | **Fluoxetine Treatment** | **Female** | **Male** |
|  | | | |
| *Activity* | control | 0.733 (0.476, 1.031) | 0.473 (0.314, 0.663) |
|  | low fluoxetine | 0.331 (0.208, 0.469) | 0.539 (0.353, 0.762) |
|  | high fluoxetine | 0.576 (0.368, 0.803) | 0.782 (0.506, 1.076) |
|  | | | |
| *Stress response* | control | 0.203 (0.132, 0.281) | 0.430 (0.290, 0.606) |
|  | low fluoxetine | 0.444 (0.282, 0.618) | 0.398 (0.257, 0.559) |
|  | high fluoxetine | 0.449 (0.297, 0.616) | 0.286 (0.196, 0.389) |

**Table S4. Output from Bayesian linear mixed-effects models fitted with default priors investigating the effects of different fluoxetine treatments in females and males for activity (mean velocity, in cm per second) and stress response (freezing, in seconds).** Bold estimates with 95% credible intervals indicate fixed effects and intercept values that differ from zero. Sex (female) and treatment (control) are the reference categories for fixed effects. Random effects are presented in standard deviation (SD) units and random variances in log SD units.

| Behaviour | Predictors | Estimate (95% CI) |
| --- | --- | --- |
| *Activity* |  |  |
| Fixed effects |  |  |
|  | Intercept | 0.40 (-0.08, 0.87) |
|  | sex (male): treatment (low fluoxetine) | 0.39 (–0.28, 1.07) |
|  | sex (male): treatment (high fluoxetine) | 0.37 (–0.32, 1.07) |
|  | sex (male) | –0.23 (–0.83, 0.37) |
|  | treatment (low fluoxetine) | –0.38 (–0.99, 0.23) |
|  | treatment (high fluoxetine) | –0.27 (–0.88, 0.38) |
|  | trial | –**0.14 (**–**0.19,** –**0.08)** |
|  | refuge use | **0.23 (0.14, 0.32)** |
|  | body size | –0.20 (–0.35, –0.06) |
| Random intercepts |  |  |
|  | mesocosm | **0.28 (0.04, 0.60)** |
| *sex: treatment* | |  |
|  | female: control | **0.58 (0.26, 0.94)** |
|  | female: low fluoxetine | **0.49 (0.23, 0.81)** |
|  | female: high fluoxetine | **0.49 (0.20, 0.82)** |
|  | male: control | **0.92 (0.61, 1.35)** |
|  | male: low fluoxetine | **0.37 (0.04, 0.72)** |
|  | male: high fluoxetine | **0.42 (0.06, 0.78)** |
| Residual variances |  |  |
| *sex: treatment* | |  |
|  | female: control | –0.17 (–0.35, 0.02) |
|  | female: low fluoxetine | –**0.57 (**–**0.76,** –**0.36)** |
|  | female: high fluoxetine | –**0.29 (**–**0.47,** –**0.10)** |
|  | male: control | –**0.39 (**–**0.56,** –**0.20)** |
|  | male: low fluoxetine | –**0.32 (**–**0.51,** –**0.13)** |
|  | male: high fluoxetine | –0.14 (–0.31, 0.05) |
|  |  |  |
| *Stress response* | | |
| Fixed effects |  |  |
|  | Intercept | 0.01 (-0.68, 0.69) |
|  | sex (male): treatment (low fluoxetine) | 0.19 (–0.31, 0.69) |
|  | sex (male): treatment (high fluoxetine) | 0.33 (–0.13, 0.79) |
|  | sex (male) | –0.14 (–0.57, 0.27) |
|  | treatment (low fluoxetine) | -0.04 (–1.02, 0.91) |
|  | treatment (high fluoxetine) | –0.27 (–1.23, 0.68) |
|  | trial | **0.05 (0.00, 0.09)** |
|  | refuge use | **0.68 (0.62, 0.75)** |
|  | body size | –0.09 (–0.21, 0.02) |
| Random intercepts |  |  |
|  | mesocosm | **0.62 (0.36, 1.07)** |
| *sex: treatment* | |  |
|  | female: control | **0.33 (0.13, 0.56)** |
|  | female: low fluoxetine | **0.32 (0.02, 0.68)** |
|  | female: high fluoxetine | **0.20 (0.01, 0.47)** |
|  | male: control | **0.61 (0.37, 0.94)** |
|  | male: low fluoxetine | **0.37 (0.11, 0.64)** |
|  | male: high fluoxetine | **0.15 (0.01, 0.37)** |
| Residual variances |  |  |
| *sex: treatment* | |  |
|  | female: control | –**0.81 (**–**0.98,** –**0.60)** |
|  | female: low fluoxetine | –**0.42 (**–**0.62,** –**0.22)** |
|  | female: high fluoxetine | –**0.41 (**–**0.58,** –**0.23)** |
|  | male: control | –**0.43 (**–**0.61,** –**0.24)** |
|  | male: low fluoxetine | –**0.48 (**–**0.66,** –**0.28)** |
|  | male: high fluoxetine | –**0.64 (**–**0.80,** –**0.46)** |

**Table S5. Correlation estimates (among and within individuals) from bivariate models fitted with default priors for activity (mean velocity, in cm per second) and stress response (freezing behaviour, in seconds) across the exposure treatments (control, low, and high fluoxetine).** Estimates of correlation coefficients with 95% credible intervals are represented for each treatment. Bold values correspond to correlation coefficients whose confidence intervals do not overlap with zero.

| Correlations | Treatment | Female | Male |
| --- | --- | --- | --- |
| *Among-individual (V_A_)* | control | 0.16 (–0.71, 0.76) | 0.04 (–0.50, 0.57) |
|  | low fluoxetine | **0.70 (0.24, 0.97)** | **0.70 (0.16, 0.98)** |
|  | high fluoxetine | 0.34 (–0.73, 0.97) | 0.22 (–0.85, 0.95) |
| *Within-individual (V_W_)* | control | **0.44 (0.22, 0.63)** | 0.07 (–0.18, 0.32) |
|  | low fluoxetine | 0.05 (–0.21, 0.31) | **0.32 (0.07, 0.54)** |
|  | high fluoxetine | –0.04 (-0.29, 0.21) | 0.01 (–0.22, 0.25) |

**References**

1. Tan H, Polverino G, Martin JM, Bertram MG, Wiles SC, Palacios MM, Bywater CL, White CR, Wong BBM. 2020 Chronic exposure to a pervasive pharmaceutical pollutant erodes among-individual phenotypic variation in a fish. *Environ. Pollut.* **263**, 114450. (doi:10.1016/j.envpol.2020.114450)

2. Wiles SC, Bertram MG, Martin JM, Tan H, Lehtonen TK, Wong BB. 2020 Long-term pharmaceutical contamination and temperature stress disrupt fish behavior. *Environ. Sci. Tech.* **54**, 8072–8082. (doi:10.1021/acs.est.0c01625)

3. Bertram MG, Ecker TE, Wong BB, O’Bryan MK, Baumgartner JB, Martin JM, Saaristo M. 2018 The antidepressant fluoxetine alters mechanisms of pre-and post-copulatory sexual selection in the eastern mosquitofish (*Gambusia holbrooki*). *Environ. Pollut.* **238**, 238–247. (doi:10.1016/j.envpol.2018.03.006)
